# Pursuing Better Representations: Balancing Discriminability and Transferability for Few-Shot Class-Incremental Learning

**DOI:** 10.3390/jimaging11110391

**Published:** 2025-11-04

**Authors:** Qi Li, Wei Wang, Hui Fan, Bingwei Hui, Fei Wen

**Affiliations:** 1 National Key Laboratory of Automatic Target Recognition, College of Electronic Science and Technology, National University of Defense Technology, Changsha 410073, China; 2 Institute of Artificial Intelligence Application, Central South University of Forestry and Technology, Changsha 410004, China; 3 School of Information Science and Electronic Engineering, Shanghai Jiao Tong University, Shanghai 200240, China

**Keywords:** few-shot learning, incremental learning, representation learning, contrastive learning

## Abstract

Few-Shot Class-Incremental Learning (FSCIL) aims to continually learn novel classes from limited data while retaining knowledge of previously learned classes. To mitigate catastrophic forgetting, most approaches pre-train a powerful backbone on the base session and keep it frozen during incremental sessions. Within this framework, existing studies primarily focus on representation learning in FSCIL, particularly Self-Supervised Contrastive Learning (SSCL), to enhance the transferability of representations and thereby boost model generalization to novel classes. However, they face a trade-off dilemma: improving transferability comes at the expense of discriminability, precluding simultaneous high performance on both base and novel classes. To address this issue, we propose BR-FSCIL, a representation learning framework for the FSCIL scenario. In the pre-training stage, we first design a Hierarchical Contrastive Learning (HierCon) algorithm. HierCon leverages label information to model hierarchical relationships among features. In contrast to SSCL, it maintains strong discriminability when promoting transferability. Second, to further improve the model’s performance on novel classes, an Alignment Modulation (AM) loss is proposed that explicitly facilitates learning of knowledge shared across classes from an inter-class perspective. Building upon the hierarchical discriminative structure established by HierCon, it additionally improves the model’s adaptability to novel classes. Through optimization at both intra-class and inter-class levels, the representations learned by BR-FSCIL achieve a balance between discriminability and transferability. Extensive experiments on mini-ImageNet, CIFAR100, and CUB200 demonstrate the effectiveness of our method, which achieves final session accuracies of 53.83%, 53.04%, and 62.60%, respectively.

## 1. Introduction

With the advancement of deep learning techniques, convolutional neural networks have enjoyed tremendous success in various visual tasks [1,2]. Thanks to large-scale annotated datasets, such as ImageNet [3], models can learn robust and comprehensive feature representations from vast amounts of data [4]. Since real-world data typically arrives in the form of continuous streams, there is a growing demand for models that can continually acquire new knowledge. In this context, Class-Incremental Learning (CIL) has garnered significant attention, aiming to continuously integrate novel classes while preserving the knowledge of previous ones [5,6]. However, most CIL studies heavily rely on sufficient training data in incremental sessions. These methods often struggle when confronted with novel classes that have scarce annotated samples in practical applications [7]. To this end, Few-Shot Class-Incremental Learning (FSCIL) has emerged [8]. FSCIL presents heightened challenges, as it must rapidly acquire new concepts from extremely limited data while avoiding catastrophic forgetting of prior knowledge.

**Figure 1:**
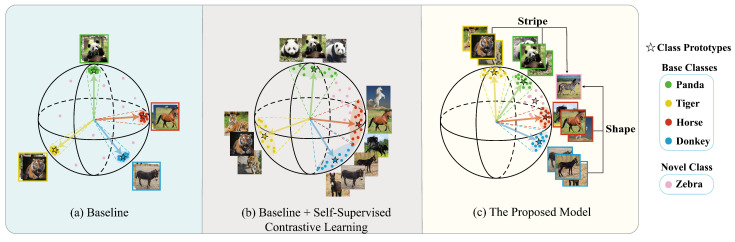
Schematic comparison of learned feature spaces. (**a**) The baseline suffers from class collapse, hindering transferability. (**b**) The introduction of SSCL harms discriminability via excessive intra-class feature spread. (**c**) Our model yields a compact, hierarchical space that optimally balances both objectives.

Early FSCIL research tended to fine-tune models on novel classes in a task-sequential manner, but it led to overfitting on novel classes and catastrophic forgetting of old ones [9,10]. To overcome these drawbacks, current mainstream FSCIL methods employ a pre-training strategy, where a powerful feature extractor (also referred to as a backbone) is trained on data-rich base classes [11]. During incremental sessions, the backbone is kept frozen to preserve learned representations, while classifier weights are replaced with normalized feature-average class prototypes. However, although this pre-training framework provides a simple yet effective approach to addressing FSCIL problems, the representations learned solely through Cross-Entropy (CE) loss training exhibit significant limitations, which fail to adapt to new tasks. As shown in Figure 1a, the CE loss compels the model to prioritize discriminative features, boosting classification accuracy but neglecting broad general information. It produces nearly identical representations for samples within the same class, a phenomenon termed class collapse [12], while such representations are highly discriminative for base classes, they struggle to transfer to novel classes, resulting in severely poor model generalization.

To address the aforementioned challenges, contemporary efforts focus on representation learning in FSCIL to improve the generalization of pre-trained models on novel classes. Building on the remarkable achievements in visual tasks, Self-Supervised Contrastive Learning (SSCL) is frequently employed in FSCIL [13,14]. Jointly optimizing both CE and SSCL losses can effectively enhance the transferability of representations. Previous work pointed out that the transferability improvement brought about by SSCL is primarily due to the spread of intra-class features [15]. Promoting feature spread implicitly contributes to the learning of more general features. Nevertheless, the SSCL paradigm for enhancing transferability exhibits significant deficiencies in the FSCIL scenario: it severely compromises the discriminability of representations. As illustrated in Figure 1b, the tactic of SSCL completely ignoring label information is in strong conflict with the CE loss. Consequently, when jointly trained with the CE loss, the pursuit of transferability in SSCL severely restricts the capture of discriminative features, leading to excessive intra-class feature spread. Excessive feature spread reduces the accuracy of class prototypes, which significantly degrades the base-class performance [16]. It is evident that learning representations with both high transferability and discriminability remains a fundamental challenge in FSCIL. Based on the above considerations, we propose a representation learning framework more suitable for the FSCIL scenario, named BR-FSCIL (Better Representations for FSCIL). BR-FSCIL is designed to learn balanced representations that maintain discriminability for base classes while enhancing transferability for novel classes through a crafted pre-training strategy.

First, we propose an innovative Hierarchical Contrastive Learning (HierCon) algorithm to avert class collapse and preserve base-class performance. Specifically, HierCon enhances feature diversity by pulling the anchor significantly closer to the augmented view of the same sample while moderately pushing away other samples from the same class in the embedding space. Simultaneously, it strongly repels features from different classes to avoid excessive spread of intra-class features. Compared to SSCL, HierCon enables modeling of hierarchical relationships among features, thereby preserving richer discriminative information when promoting transferability.

Second, as HierCon avoids excessive intra-class spread, the model generalization for novel classes is marginally insufficient. To address this deficiency, we further introduce an Alignment Modulation (AM) loss. The AM loss is designed to promote the learning of transferable knowledge across tasks by aligning the average activation of key feature patterns among different classes. Moreover, instead of directly promoting intra-class feature spread, it reduces divergence of key patterns across classes. This operation explicitly enhances transferability from an inter-class perspective while maintaining intra-class similarity to preserve discriminability. Figure 1c visually illustrates the feature space obtained by the BR-FSCIL model. HierCon constructs a hierarchical feature structure in which features within the same class are moderately spread, while boundaries between classes remain distinct. The AM loss explicitly facilitates the learning of inter-class shared features—such as the striped patterns common to tigers and pandas, or morphological traits shared by horses and donkeys. These shared features collectively form the discriminative basis for novel classes (e.g., zebras), thereby enhancing the model’s adaptability. Critically, the AM loss does not compromise intra-class compactness; on the contrary, it enhances transferability while preserving hierarchical discriminative structures by reducing inter-class distance. Together, HierCon and the AM loss synergistically construct a compact yet hierarchically structured feature space, simultaneously achieving high discriminability and transferability.

After pre-training, the BR-FSCIL model parameters are frozen and employ the nearest class mean classifier [17] for incremental adaptation. Extensive experiments demonstrate that BR-FSCIL greatly enhances model generalization for novel classes while maintaining discrimination for base classes. Compared with existing methods, our method offers superior adaptability and performance in the FSCIL task.

Our contributions can be summarized as follows.

- We identify that the core challenge for pre-training-based FSCIL methods lies in balancing the discriminability and transferability of the learned representations. To address this issue, we propose a novel representation learning framework for FSCIL.
- During the pre-training stage, we first propose a HierCon algorithm to alleviate class collapse while preserving strong base-class performance. We then incorporate the AM loss to further enhance representation transferability by facilitating the learning of knowledge shared across classes. The synergistic action of HierCon and the AM loss yields learned representations that ensure high transferability and discriminability.
- Extensive experiments on three standard FSCIL benchmarks show that our method provides a more effective solution for the FSCIL scenario, which achieves final session accuracies of 53.83% on mini-ImageNet, 53.04% on CIFAR100, and 62.60% on CUB200.

## 2. Related Works

This section reviews related works encompassing Class-Incremental Learning, Few-Shot Class-Incremental Learning, and Representation Learning. Key information from all relevant studies has been summarized in Table 1. Additionally, we have deliberately clarified the differentiation of our BR-FSCIL from prior works to directly showcase its innovative contributions.

### 2.1. Class-Incremental Learning

CIL aims to integrate knowledge from novel classes while maintaining the ability to recognize previously learned ones. Conventional CIL methods can be broadly categorized into three paradigms: regularization, replay, and knowledge distillation techniques. Regularization methods assess the importance of network parameters and mitigate catastrophic forgetting by imposing constraints on those deemed critical. EWC [18] utilizes the Fisher Information Matrix to protect significant parameters during updates. SOUL [19] reduces inter-class confusion within the Bi-GCN classifier. Replay methods store samples from old classes and replay them during incremental phases to alleviate catastrophic forgetting. iCaRL [20] adopts a class-prototype-based sample selection strategy to mitigate forgetting. AAER [21] employs anchors as representative samples of prior classes. Knowledge distillation methods aim to align the current model’s outputs with those of the previous model to preserve consistency in knowledge representation. LwF [22] mitigates catastrophic forgetting when learning new concepts by employing a distillation loss to constrain shifts in the output space. EEIL [23] introduces an end-to-end framework for incremental learning. In recent years, the advancement of large-scale pre-trained models has sparked growing interest in fine-tuning methodologies. Such methods improve the transferability of pre-trained Vision Transformer (ViT) [24] models to unseen classes by incorporating tailored visual prompting mechanisms. For instance, LRT [25] rapidly adapts to novel objects by leveraging visual cues and textual descriptions from a pre-trained large model.

With the advancement of deep learning, CIL methods have significantly mitigated the problem of catastrophic forgetting. Yet, they typically require substantial data in each incremental session and underperform in data-scarce scenarios. Therefore, the FSCIL problem we investigate holds greater practical significance.

### 2.2. Few-Shot Class-Incremental Learning

Recently, FSCIL has been proposed to address the limitations of CIL in data-scarce scenarios. FSCIL aims to continuously learn novel classes from limited samples while preserving recognition capability for old classes. TOPIC [8] firstly introduces the FSCIL task, whose method still involves fine-tuning during the incremental stage. Since CEC [11] demonstrates the effectiveness of the decoupled framework (i.e., a fixed feature extractor coupled with a non-parametric classifier), research in this field has primarily focused on pre-training algorithms. For example, FACT [26] generates pseudo novel-class samples through mix-up augmentation and compresses the feature space to accommodate novel classes. SAVC [27] adopts a more aggressive augmented supervised contrastive learning strategy, leading to substantial gains in base-class performance. CLOSER [16] balances base and novel class performance by reducing inter-class distances. BFCP [28] focuses on enhancing the forward compatibility of pre-trained models.

Unlike methods that introduce complex fine-tuning mechanisms, these pre-training-based methods not only effectively mitigate overfitting on novel classes but also offer computational efficiency. Similarly, this paper concentrates on optimizing the model’s representational capabilities during the pre-training stage. Our objective is to develop balanced representations that simultaneously achieve high discriminability and transferability.

### 2.3. Representation Learning

Representation learning, a core machine learning field, develops algorithms to extract comprehensive features from raw data for downstream tasks. It has gained significant attention in pre-training-based FSCIL, where models trained solely on base sessions require transferable representations for the incremental phase. Several studies have explored transferable representation learning, including the use of lower temperature [29] and negative margins [30] in the softmax cross-entropy loss to enhance the transferability of representations. Moreover, contrastive learning has gained prominence as a powerful approach for representation learning, while Supervised Contrastive Learning (SupCon) [31] still grapples with the issue of class collapse, Self-Supervised Contrastive Learning (SSCL), such as SimCLR [32], has proven effective in enhancing downstream adaptability [33]. AsyCLR [7] introduces predictive features to achieve asymmetric alignment of positive pairs, thereby mitigating excessive similarity within positive features. Nevertheless, these methods are not fully compatible with the FSCIL scenario. Existing research revealed that the key to the transferability gains of these methods lies in intra-class feature spread. CLOSER [16] further highlights the negative impact of excessive feature spread on base-class performance. Additionally, CLOSER finds that smaller inter-class distances can substantially benefit transferability, providing valuable insight for our research.

In light of this, we aim to develop a representation learning framework better suited for FSCIL. Unlike methods that sacrifice base-class performance for gains on novel classes, our method aims to learn balanced representations that maintain strong discriminability while possessing high transferability.

### 2.4. Distinctions from Related Representation Learning Frameworks

To highlight the uniqueness and innovation of our BR-FSCIL, we provide a systematic elaboration here on how our approach differentiates itself from existing representation learning frameworks commonly used in FSCIL, such as SSCL, SupCon, CLOSER, and AsyCLR.

Traditional SSCL frameworks disregard inter-class distinctions, while they can acquire highly transferable representations, this comes at the cost of severely compromising the model’s discriminability on source data, rendering them unsuitable for FSCIL scenarios. In contrast, inspired by SupCon, the HierCon in our BR-FSCIL incorporates label information to mitigate the damage to discriminability when enhancing transferability. Although both HierCon and SupCon align the same-class features, SupCon treats all in-class samples equally as positives. Our HierCon; however, innovatively introduces a hierarchical structure that distinguishes between “strong positives” (augmented views) and “weak positives” (other in-class samples). This distinction better models hierarchical relationships among features, thereby enhancing representation transferability while avoiding the detrimental effect of excessive feature spread on base-class performance.

BR-FSCIL further employs the AM loss to advance transferable feature learning. The AM loss implicitly encourages a more compact feature space, which is inherently more conducive to transferability, while this shares a similar underlying intuition with CLOSER regarding compactness, their implementations are fundamentally different. CLOSER globally compresses the feature space to reduce inter-class distances. In comparison, our AM loss is more selective, aligning only the most critical feature channels across classes. This targeted approach better preserves the discriminative structure of the feature space while enhancing transferability.

Finally, AsyCLR focuses on asymmetric learning within the same class by introducing predictive features to alleviate class collapse. In contrast, our HierCon is symmetric and leverages original features to explicitly model the hierarchical inter-class structure. More significantly, our AM loss introduces a new mechanism operating on class-level feature statistics, representing a fundamental departure from AsyCLR’s methodology.

In summary, our BR-FSCIL effectively addresses the core challenge of balancing discriminability and transferability in FSCIL through the synergistic interaction between HierCon’s hierarchical contrast and AM loss is selective alignment. By transcending the limitations of existing paradigms, our work establishes a more precise and effective pathway for representation learning in incremental scenarios.

## 3. Materials and Methods

**Figure 2:**
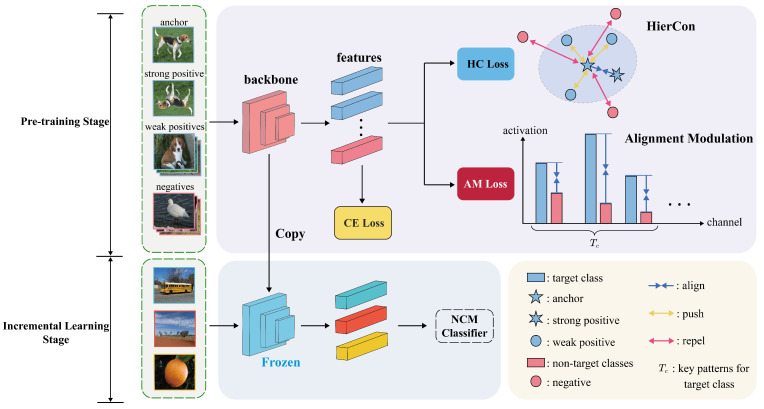
Framework of BR-FSCIL. During the pre-training stage, we jointly optimize the model with the HierCon algorithm and AM loss in addition to the CE loss. More details are discussed in Section 3. After pre-training, the backbone remains frozen throughout subsequent incremental sessions, and the NCM classifier is used for classification.

The overall framework of BR-FSCIL comprises two primary stages: pre-training and incremental learning, as depicted in Figure 2. To clearly present the principles and motivation of the proposed method, this section elaborates on the problem definition of FSCIL, the limitations of existing methods, and the implementation details of our method.

### 3.1. Problem Definition

FSCIL consists of a base session and several sequentially arriving incremental sessions. Crucially, the model is only exposed to the current training data in each session, yet evaluated on all classes seen so far. Formally, the training datasets for these sessions can be denoted as: $\left\{D_{train}^{0},D_{train}^{1},\ldots ,D_{train}^{M}\right\}$. The base stage, denoted as $D_{train}^{0}$, contains a sufficient amount of labeled data for training. Subsequent sessions are denoted as $D_{train}^{m}$, and the training data follows the N−wayK−shot configuration, i.e., *N* classes, each providing exactly *K* labeled training samples. The label space corresponding to the training dataset $D_{train}^{m}$ is denoted by $C^{m}$. It is noteworthy that the label spaces of the training sets across all phases are mutually disjoint, i.e., ∀i,j and $i\neq j,C^{i}\cap C^{j}=⌀$. The label space of the test dataset $D_{test}^{m}$ encompasses all classes encountered in both the current session and all previous sessions, i.e., $C^{0}\cup C^{1}\ldots \cup C^{m}$.

### 3.2. Limitations of Existing Methods

#### 3.2.1. Baseline

A classification agent can be formulated as comprising a backbone $f_{\theta }\left(\cdot \right)$ with parameters θ and a classifier with weights *w*. During the base session, it is trained using the Cross-Entropy (CE) loss, with logits defined by the cosine similarity between features and classifier weights [34]:

(1) $$L_{\mathrm{CE}}^{i}=-\log\;\;\frac{\exp\;\;(\frac{1}{\tau }\mathrm{sim}\left.\;\;(\left.f_{\theta }\left(x_{i}\right),w_{c}\right)\right)}{\sum_{j=1}^{\left|C^{0}\right|}\exp\;\;(\frac{1}{\tau }\mathrm{sim}\left.\;\;(\left.f_{\theta }\left(x_{i}\right),w_{j}\right)\right)},$$

where $\mathrm{sim}\left(\cdot ,\cdot \right)$ denotes cosine similarity, τ is the temperature parameter and $w_{c}$ represents the classifier’s weight vector corresponding to the ground-truth class of sample $x_{i}$.

To avoid catastrophic forgetting and overfitting caused by fine-tuning on limited novel-class samples, the baseline keeps the pre-trained backbone frozen during incremental sessions. At this moment, classification is performed using the non-parametric normalized Nearest Class Mean (NCM) [17] classifier. The classifier weight for class *c* is calculated by normalizing the feature-average class prototype of its training samples:

(2) $$w_{c}=\frac{1}{N_{c}}\sum_{x_{i},y_{i}\in D_{train}^{m}}I_{\left(y_{i}=c\right)}\frac{f_{\theta }\left(x_{i}\right)}{\;\;{∥f_{\theta }\left(x_{i}\right)∥}_{2}},$$

where $N_{c}$ is the number of samples in class *c*, and I is the indicator function that equals 1 if the subscript condition is satisfied and 0 otherwise.

**Figure 3:**
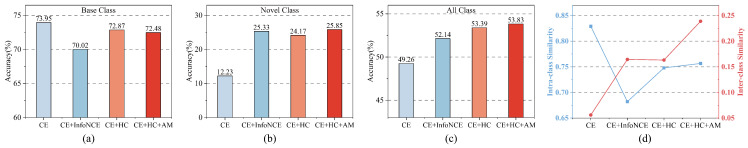
Performance comparison of different loss functions in the final session on Mini-ImageNet. (**a**) Our method effectively mitigates base-class performance degradation. (**b**) Our method achieves significant advantages in novel class recognition. (**c**) Our method yields the best overall performance. (**d**) Our method simultaneously optimizing intra-class and inter-class similarity.

From Figure 3, although the backbone trained by the baseline demonstrates strong performance on base classes, its generalization to novel classes is extremely weak. The results in Figure 3d indicate that the phenomenon is primarily attributed to excessively similar intra-class representations. Excessive intra-class similarity results in poor transferability of representations. We confirm that the CE loss leads to class collapse.

#### 3.2.2. Self-Supervised Contrastive Learning

Due to its strong performance in visual tasks, Self-Supervised Contrastive Learning (SSCL) is widely adopted in FSCIL. Existing methods, largely based on SimCLR [32], use an augmented view of the query sample as positive and all other samples as negatives. SSCL leverages the InfoNCE loss [35] to push positive pairs together while repelling negative pairs:

(3) $$L_{\mathrm{InfoNCE}}^{i}=-\log\frac{\exp(\frac{1}{\tau }\mathrm{sim}\left(z_{i},z_{j}\right))}{\sum_{k=1}^{N}I_{\left(k\neq i\right)}\exp\left(\frac{1}{\tau }\mathrm{sim}\left(z_{i},z_{k}\right)\right)},$$

where *N* denotes the number of samples including augmented instances, $z_{j}$ represents the feature from an augmented view of $x_{i}$.

Jointly optimizing the CE loss and InfoNCE loss improves the performance on novel classes, as demonstrated in Figure 3b. SSCL’s approach of uniformly distributing different instance features across the hypersphere promotes intra-class feature spread, which facilitates the learning of transferable features. However, Figure 3a suggests that the improvement in performance on novel classes comes at the cost of reduced discriminative ability on base classes. According to the results in Figure 3d, the decline in base-class performance is attributed to a significant decrease in intra-class similarity, which severely undermines the precision of class prototypes. The underlying cause lies in the fact that SSCL’s optimization objective completely ignores class label information, leading to a strong conflict with the CE loss. Consequently, SSCL’s pursuit of high transferability considerably hinders the model’s ability to capture discriminative information, resulting in excessive intra-class feature spread and ultimately significantly reducing intra-class similarity. Moreover, the trade-off reveals a fundamental limitation of previous methods in FSCIL: relying solely on promoting feature spread to enhance representation transferability inevitably sacrifices its discriminability, thereby trapping the model in the stability–plasticity dilemma.

### 3.3. Hierarchical Contrastive Learning

Based on the preceding analysis, SSCL’s disregard for label information severely degrades base-class performance. The shortcoming makes it difficult to achieve an optimal balance between discriminability and transferability. In contrast, although SupCon [31] cannot enhance representation transferability, it effectively leverages label information to align intra-class features [7]. Given an anchor feature $z_{a}$, SupCon treats features from the same class as positives and those from other classes as negatives:

(4) $$L_{\mathrm{SupCon}}^{a}=-\frac{1}{\left|P(a)\right|}\sum_{z_{p}\in P\left(a\right)}\log\frac{\exp(\frac{1}{\tau }\mathrm{sim}\left(z_{a},z_{p}\right))}{\sum_{z_{i}\in I\left(a\right)}\exp(\frac{1}{\tau }\mathrm{sim}\left(z_{a},z_{i}\right))},$$

where $P\left(a\right)$ denotes the set of positive features for $z_{a}$, and $I\left(a\right)$ represents other features in the mini-batch excluding $z_{a}$.

Inspired by SupCon, we propose a novel Hierarchical Contrastive Learning (HierCon) algorithm to prevent class collapse while maintaining robust base-class performance. Critically, building upon SSCL, HierCon incorporates category information to model hierarchical relationships among features. It not only achieves instance-level discrimination but also maintains inter-class separation. Detailed calculation steps follow.

As depicted in Figure 2, following the SimCLR [32] framework, we apply two stochastic data augmentation to each image within a batch of base-class samples. Each input image thus produces two randomly augmented views. For the anchor feature $z_{a}$, the feature from another augmented view of the same image is regarded as the strong positive, denoted as $z_{s}$. Features from other images belonging to the same class are considered weak positives, denoted as the set $W\left(a\right)$. Furthermore, features from all images of other classes are treated as negatives. The loss function of HierCon (HC loss) is designed to pull the anchor closer to the strong positive, keep a reasonable distance from weak positives, and significantly repel negatives. This helps disperse features within the same class while maintaining separation between different classes. In line with SSCL, we project all features onto a unit hyper-sphere to enhance training stability. The HC loss for the anchor feature $z_{a}$ is defined as follows:

(5) $$L_{\mathrm{HC}}^{a}=\alpha L_{\mathrm{strong}}^{a}+\left(1-\alpha \right)L_{\mathrm{weak}}^{a},$$

where

(6) $$L_{\mathrm{strong}}^{a}=-\log\frac{\exp(\frac{1}{\tau }\mathrm{sim}\left(z_{a},z_{s}\right))}{\sum_{z_{i}\in I\left(a\right)}\exp(\frac{1}{\tau }\mathrm{sim}\left(z_{a},z_{i}\right))},$$

(7) $$L_{\mathrm{weak}}^{a}=-\frac{1}{\left|W(a)\right|}\sum_{z_{w}\in W\left(a\right)}\log\frac{\exp(\frac{1}{\tau }\mathrm{sim}\left(z_{a},z_{w}\right))}{\sum_{z_{i}\in I\left(a\right)}\exp(\frac{1}{\tau }\mathrm{sim}\left(z_{a},z_{i}\right))},$$

The HC loss comprises two parts: strong and weak. Strong facilitates alignment between anchor and strong positive while uniformly repelling all other samples to spread intra-class features. Weak incorporates label information to counteract the over-dispersion of intra-class features resulting from the strong. By balancing the contributions of strong and weak components through a coefficient $\alpha \in \left(0,1\right]$, HierCon effectively distinguishes between strong and weak positives, thereby constructing a feature space with multi-level semantic structures. Notably, our HierCon diverges from established contrastive learning frameworks (e.g., SimCLR) by omitting the non-linear projection head that typically maps feature vectors to an alternate space. This design stems from the requirement for joint optimization between the HC loss and other loss functions. Introducing a projection head would decouple the feature spaces: the HC loss would operate on the projected feature space while other losses would act on the original feature space. This spatial misalignment would hinder synergistic optimization across the different objectives and ultimately degrade model performance, as confirmed by our ablation study on the non-linear projection head.

As presented in Figure 3, joint optimization using both CE loss and HC loss effectively reduces intra-class similarity, thereby preventing the class collapse. Moreover, although HierCon shows slightly weaker generalization to novel classes compared to SSCL, it significantly alleviates the performance degradation on base classes. This implies that HierCon and CE loss exhibit improved compatibility. To provide a more comprehensive comparison between HierCon and SSCL, we further present their performance with different loss weights in subsequent experiments. Experimental results clearly demonstrate that HierCon preserves richer discriminative information while promoting feature spread. It effectively mitigates the degradation of discriminability resulting from the pursuit of transferability, leading to a more optimal balance.

### 3.4. Alignment Modulation

Compared to SSCL, HierCon achieves a better performance balance due to the more rational feature spread. However, it is slightly inferior in model generalization. Moreover, relying exclusively on intra-class feature spread for improving transferability is not entirely applicable to FSCIL. To further enhance transferability while preserving discriminability, we incorporate a structured constraint from a pattern recognition perspective to explicitly guide the model toward learning more transferable features. Studies on deep network interpretability indicate that individual feature channels can be viewed as fundamental semantic patterns [36,37]. Together, these patterns form the basis of categorical discrimination [38]. Different classes express their distinctiveness through specific combinations of these patterns. The activation intensity reflects how much a sample aligns with a particular pattern.Since semantic correlations exist across classes (e.g., pandas, zebras, and tigers share similar stripe patterns), some patterns activated in one class are also activated in others. We refer to these as transferable patterns. The effective representations should comprise abundant cross-class transferable patterns, which can not only support discrimination within known classes but also be effectively adapted to represent unknown classes. Motivated by this insight, we propose an Alignment Modulation (AM) loss to explicitly encourage the model to learn more transferable patterns.

Specifically, let $p_{t}\left(x_{i}\right)$ denote the activation of the *t*-th channel in the feature vector of sample $x_{i}$. The average activation of the *t*-th channel for all feature vectors of class *c* is as follows:

(8) $$\mu_{c,t}=\frac{1}{N_{c}}\sum_{x_{i},y_{i}\in D_{train}^{0}}I_{\left(y_{i}=c\right)}p_{t}\left(x_{i}\right),$$

similarly, we can calculate the average activation of the *t*-th channel for all sample feature vectors not belonging to class *c*:

(9) $$\mu_{\bar{c},t}=\frac{1}{N_{\bar{c}}}\sum_{x_{i},y_{i}\in D_{train}^{0}}I_{\left(y_{i}\neq c\right)}p_{t}\left(x_{i}\right),$$

where $N_{\bar{c}}$ is the number of the samples not belonging to class *c*. We utilize the classifier weights corresponding to class *c* to select the top-weighted feature channels for the category, which are defined as the set of key patterns, denoted as $T_{c}$. Since transferable patterns can be activated across different classes, we enhance the transferability of representation by aligning the activations of these key patterns between classes. This is formalized as follows:

(10) $$L_{\mathrm{AM}}=\frac{1}{\left|C^{0}\right|}\sum_{c\in C^{0}}\frac{1}{\left|T_{c}\right|}\sum_{t\in T_{c}}{\left(\mu_{c,t}-\mu_{\bar{c},t}\right)}^{2}.$$

In contrast to SSCL and HierCon, the AM loss does not directly promote intra-class feature spread to preserve discriminability. Instead, it essentially enhances the learning of knowledge shared across classes from an inter-class perspective, which can be effectively transferred to new tasks. Figure 3 supports our claim. When the AM loss is jointly optimized with both the CE and HC losses, the model achieves significantly improved classification accuracy on novel classes while maintaining strong performance on base classes. From the perspective of feature relationships, Figure 3d shows that the intra-class similarity remains largely unchanged after introducing the AM loss. This indicates that the loss function effectively preserves the hierarchical feature structure constructed by HierCon, thereby maintaining base-class performance. Conversely, the AM loss increases inter-class similarity, resulting in a more compact feature space. Our findings align with the view presented in CLOSER [16] that a compact feature space facilitates the transferability of representations while maintaining discriminability. Consequently, the compact yet semantically hierarchical feature space, jointly constructed by HierCon and the AM loss, maintains the discriminability of base classes while enhancing the transferability of representations and reserving capacity for novel classes. Our emphasis on feature space compactness is similar to CLOSER. However, unlike CLOSER’s approach, which requires all features across different classes to be similar, the AM loss enables more precise learning of cross-task transfer knowledge through targeted pattern alignment, leading to a better balance.

Accordingly, our pre-training strategy aims to minimize the following loss:

(11) $$O=\arg\;\;\min_{\theta ,w}\left[L_{\mathrm{CE}}\left(D_{train}^{0};\theta ,w\right)+\lambda L_{\mathrm{HC}}\left(D_{train}^{0};\theta \right)+\gamma L_{\mathrm{AM}}\left(D_{train}^{0};\theta \right)\right],$$

where λ and γ are hyper-parameters that balance the different losses. Following the baseline, we conduct an incremental evaluation using the normalized NCM classifier.

## 4. Results and Analysis

This section presents the experimental results of BR-FSCIL and provides a detailed analysis.

### 4.1. Experimental Setup

#### 4.1.1. Datasets

Our study employs three benchmark datasets, namely mini-ImageNet [39], CIFAR100 [40], and CUB200 [41], based on the following rationales. First, they are widely recognized as standard benchmarks in the FSCIL. The majority of existing studies adopt these datasets [8,11], which ensures fair and direct comparison between our experimental results and those of mainstream approaches. Second, these three datasets offer complementary characteristics in terms of task focus: mini-ImageNet and CIFAR100 provide relatively complex natural images, while CUB200, as a fine-grained dataset, imposes higher demands on the model’s discriminative capability. Such a diverse combination allows for a comprehensive evaluation of the proposed method’s effectiveness and robustness across different scenarios.

The three standard datasets adopted in this study exhibit the following characteristics and distinctions. The mini-ImageNet dataset consists of 60,000 color images from 100 classes, with each class containing 600 images of 84 × 84 pixel resolution. Following standard literature settings [8,11], we select 60 classes as base classes. The remaining 40 novel classes are further divided into eight incremental sessions under a typical five-way five-shot experimental setup. The CIFAR100 dataset comprises 100 classes, each providing 600 color images of 32 × 32 pixels, with 500 for training and 100 for testing. For FSCIL, the split for CIFAR100 is identical to that of mini-ImageNet. The CUB200 dataset is a fine-grained dataset focused on bird species. It contains 11,788 color images of 224 × 224 pixel resolution, covering 200 bird classes. The dataset is split into 100 base classes and 100 novel classes, with the latter divided into 10 incremental sessions, each containing 10 classes in a 10-way 5-shot format. The detailed statistics of the three datasets are listed in Table 2.

As described, FSCIL datasets are inherently imbalanced—base classes contain abundant samples while each novel class has only a few. This imbalance severely impacts model performance by exacerbating catastrophic forgetting of base classes and leading to overfitting to novel classes, posing a distinctive challenge in FSCIL. Accordingly, our BR-FSCIL framework is designed to counteract the effects of this inherent imbalance. By deriving a powerful feature backbone through well-designed base-session pre-training and keeping it frozen during incremental sessions, our approach effectively addresses the data imbalance challenge in FSCIL (see detailed experimental results in Section 4.2).

#### 4.1.2. Implementation Details

Following previous works [11], we use the ResNet as the backbone. Specifically, we employ ResNet-20 for CIFAR100, and ResNet-18 for both mini-ImageNet and CUB200. For CUB200, the ResNet-18 is pre-trained on ImageNet-1k [3]. We set the batch sizes for mini-ImageNet, CIFAR100, and CUB200 to 128, 128, and 256, respectively. During the pre-training stage, we use an SGD optimizer with a momentum of 0.9 and a weight decay of $5\times 10^{-4}$. We train for 400 epochs on CIFAR100 and mini-ImageNet with an initial learning rate of 0.1, and for 100 epochs on CUB200 with an initial learning rate of 0.05. All learning rates follow a half-cosine annealing schedule. Image augmentation techniques include random cropping, horizontal flipping, AutoAugment, and normalization. Notably, AutoAugment is not applied to the CUB200 dataset due to its color sensitivity. The optimal hyper-parameters, determined through hyper-parameters ablation studies on the datasets, are set as follows: on mini-ImageNet and CIFAR100, λ=0.3, γ=0.5, α=0.9, and $|T_{c}|=3$; on CUB200, λ=0.1, γ=1.0, α=0.9, and $|T_{c}|=8$. During training, $T_{c}$ is updated dynamically in sync with the classifier parameters on a per-batch basis. Additionally, we set the training temperature τ to 1/16. All experiments are implemented using the PyTorch (version 2.2.2) framework on a single NVIDIA RTX 4090 GPU (from NVIDIA Corporation, Santa Clara, CA, USA).

#### 4.1.3. Evaluation Protocol

Consistent with CEC [11], we evaluate model performance using the top-1 accuracy after each session and the performance dropping rate (PD), defined as the drop in accuracy from the initial session to the final session. Additionally, we report the average accuracy (Avg) across all incremental sessions.

### 4.2. Comparison with SOTAs

We compare our proposed BR-FSCIL with state-of-the-art methods on three benchmark datasets. These methods primarily include classical CIL method: iCaRL [20]; pre-training-based FSCIL methods: CEC [11], FACT [26], SAVC [27], CLOSER [16], and BFCP [28]; fine-tuning-based FSCIL methods: TOPIC [8], SoftNet [42], UaD-CE [43], and LIMIT [44]. For the convenience of subsequent experiments, we reproduce the results for FACT and CLOSER using their publicly available codes. The detailed results are presented in Table 1, Table 2 and Table 3.

Table 3 shows that on the mini-ImageNet dataset, BR-FSCIL achieves the best final performance, outperforming the second-best method by 1.12%, while our accuracy is slightly lower than SAVC in the first two sessions, this is because SAVC over-prioritizes base class performance. It leads to a substantial drop in performance during the incremental phase due to a lack of generalization. In contrast, our method demonstrates superior adaptability in incremental sessions, achieving a performance dropping rate of 23.49%. Despite a slightly higher dropping rate compared to LIMIT, our method avoids the inevitable decline in base-class performance that results from fine-tuning LIMIT on novel classes. Thus, our method achieves the highest average accuracy of 63.64% across all sessions. These results highlight the unique advantages of the proposed method: it not only maintains strong discriminability for base classes but also enhances transferability for novel classes.

On the CIFAR100 and CUB200 datasets, as shown in Table 4 and Table 5, BR-FSCIL also demonstrates strong performance. Specifically, it consistently achieves first or second-best results across individual sessions. Our method delivers average accuracy rates of 63.08% and 68.58% across all sessions on CIFAR100 and CUB200, respectively, outperforming the second-best method by margins of 1.19% and 0.47% in the final session. Notably, the performance gain of our method on the CUB200 dataset is not as significant as on the other two. This could be because the inherent fine-grained nature of CUB200 requires the model to focus more on local discriminative features, which reduce the advantage of our method.

### 4.3. Ablation Study

In this subsection, we evaluate the effectiveness of each component—including the CE loss, HC loss, and AM loss—on mini-ImageNet. To quantify the specific contribution of each component, we present the base ($Acc_{B}$) and novel ($Acc_{N}$) class accuracies in the final session. Detailed results of the ablation study are presented in Table 6.

Table 6 reveals that utilizing the CE loss alone leads to outstanding performance on base classes in the final session, but results in a mere 12.23% accuracy on novel classes, demonstrating catastrophic forgetting. By incorporating the HC loss, the novel-class accuracy significantly improves by 11.94%, while base-class performance only drops by 1.08%. This represents a much smaller decline in base-class performance than that caused by SSCL (1.08% vs. 3.93%), indicating that the HC loss not only prevents catastrophic forgetting but also preserves discriminability for base classes. Furthermore, the incorporation of the AM loss enhances the model’s classification performance across all training sessions. Although a minor degradation in base-class accuracy is observed in the final session (a decrease of less than 0.5%), the recognition performance on novel classes improves by 1.68%. This demonstrates that the AM loss enables the model to learn more transferable patterns, thereby facilitating its adaptability to novel classes. It is noteworthy that while combining either the HC loss or the AM loss with the CE loss individually leads to performance improvement, it does not yield the optimal result. These findings indicate that although both HierCon and AM losses individually enhance representation quality, their effects are not redundant. Only through the synergistic interaction between HierCon and AM losses can the model achieve optimal performance.

Additionally, we experimented with excluding the CE loss and training solely with HC or AM losses, which yielded suboptimal results, while the HC loss focuses on inter-class differences, it fails to enforce strong intra-class feature alignment, leading to severely compromised base-class performance and, consequently, substantial degradation of representation quality. Meanwhile, the AM loss lacks intra-class alignment, rendering the model unable to learn effective representations for each class. This evidence suggests that the HC and AM losses serve as auxiliary components to the CE loss, providing a more comprehensive representation. Only through the collaborative operation of all three losses can the model simultaneously learn representations that are both high in discriminability and transferability.

### 4.4. Analysis of Hyper-Parameters

**Figure 4:**
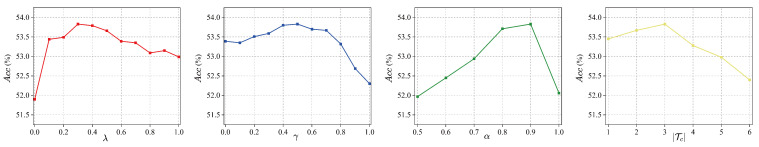
Impact of the four hyper-parameters on mini-ImageNet. When varying one hyper-parameter, the other three are held fixed.

Our BR-FSCIL involves four key hyper-parameters: the weighting coefficients λ, γ, and α in Equations (5) and (11), and $\left|T_{c}\right|$ in (10). To thoroughly investigate the impact of these hyper-parameters on model performance, we report the classification accuracy in the final session (Acc) under different hyper-parameter settings on the mini-ImageNet dataset. Based on the results presented in Figure 4, the model achieves its best performance across all classes when λ, γ, and α, are set to 0.3, 0.5, and 0.9, respectively. It indicates that either excessively high or low loss weights can disrupt the balance between the discriminability and transferability of the learned representations. Furthermore, the model attains optimal performance when the number of key patterns $\left|T_{c}\right|$ is set to 3. Too few patterns provide insufficient model generalization improvement, while too many patterns harm the discrimination for base classes. Given that the category setup of the CIFAR100 dataset is highly similar to that of mini-ImageNet, we keep the hyper-parameter settings consistent with those used for mini-ImageNet. Experimental results in Table 4 demonstrate the effectiveness of this hyper-parameter configuration on CIFAR100.

**Figure 5:**
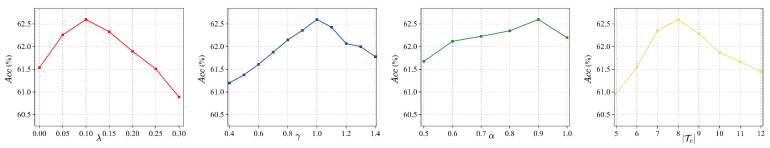
Impact of the four hyper-parameters on CUB200. When varying one hyper-parameter, the other three are held fixed.

In addition, since the fine-grained characteristics of CUB200 differ substantially from those of the other two datasets, we also examine the impact of different hyperparameters on model performance on CUB200. As shown intuitively in Figure 5, CUB200 exhibits higher sensitivity to λ, which can be attributed to its fine-grained features, leading the model to focus more on local discriminative properties and thereby influencing the optimization of the HC loss. Consequently, the optimal value for λ on CUB200 is only 0.1. With HC loss playing a less dominant role, the optimal value of α remains relatively stable. Similarly, the fine-grained nature of CUB200 results in abundant shared features among classes; thus, the optimal values for γ and $\left|T_{c}\right|$ are larger than those on mini-ImageNet. Ultimately, the model achieves optimal performance on CUB200 when λ, γ, α, and $\left|T_{c}\right|$ are set to 0.1, 1.0, 0.9, and 8, respectively.

### 4.5. Analysis of HierCon

#### 4.5.1. Effect of Non-Linear Projection Head

When combined with other loss functions, our HierCon algorithm does not employ a non-linear projection head ike other contrastive learning methods. To study the effect of a non-linear projection head, we added a single-hidden-layer MLP *g* (hidden dimension 512, output dimension 128) to HierCon. It projects features from the backbone into a new space. Incorporating the projection head *g* into HierCon results in a significant degradation in overall performance, according to the results presented in Table 7. We attribute this to the projection head *g* causing the HC loss and other losses to operate in divergent feature spaces, which adversely affects the overall performance. This observation validates the rationality of omitting the non-linear projection head in our design.

#### 4.5.2. Comparison with SSCL

**Figure 6:**
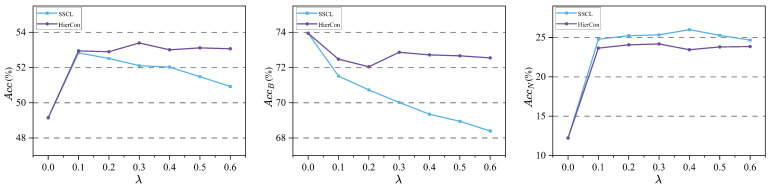
Comparison of contrastive learning algorithms under different loss weights.

To comprehensively compare HierCon and SSCL, we demonstrate the performance of both contrastive learning losses under different weight λ when jointly optimized with CE loss. The SSCL framework remains based on SimCLR, and similarly omits the non-linear projection head. The subfigures in Figure 6 display the overall, base-class, and novel-class classification accuracy of each algorithm in the final session. As demonstrated by our results, increasing the weight of SSCL loss yields moderate gains in novel-class performance, but at the expense of a significant decline in base-class accuracy, ultimately degrading overall performance. It confirms that SSCL’s pursuit of better transferability compromises its discriminative capability for base classes. In contrast, although HierCon achieves slightly lower gains on novel classes, it maintains strong base-class performance even with increasing weights, resulting in a better trade-off.

### 4.6. Analysis of AM Loss

Here, we employ multiple methods to reduce discrepancies among key inter-class patterns. Specifically, the average activations of key patterns from the target class and those corresponding to the same patterns in non-target classes are treated as a pair of vectors. These vectors are aligned using L1 loss, Cosine Similarity loss, and KL Divergence loss. Additionally, we implement the method from CLOSER [16], which directly increases the cosine similarity between features of all different classes to reduce inter-class distance, denoted as Inter loss. As presented in Table 8, L1, Cosine Similarity, and KL Divergence losses yield only marginal gains in representation transferability, leading to suboptimal model generalization to novel classes. Although Inter loss significantly enhances the model generalization, excessive feature similarity can impair the discriminative power for base classes. Our proposed AM loss outperforms all compared strategies, demonstrating the best balance between base-class and novel-class performance.

### 4.7. Further Analysis

#### 4.7.1. Prediction Bias

**Figure 7:**
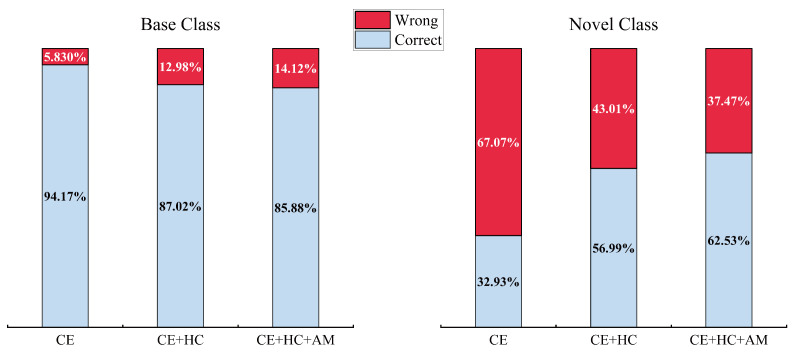
Prediction bias between base and novel classes.

For a basic binary classification task (i.e., simply categorizing test samples into base and novel classes), the baseline trained solely with the CE loss exhibits a significant prediction bias, as illustrated in Figure 7, while it achieves relatively high accuracy on base classes, its error rate on novel classes reaches as high as 67%. The results indicate severe overfitting to the base classes and a notable lack of generalization to novel classes. In contrast, the joint optimization method, incorporating the proposed HC and AM losses, significantly reduces the error rate on novel classes to 37.47%, effectively improving classification balance. Our results confirm that BR-FSCIL mitigates prediction bias between base and novel classes, resulting in more balanced predictions.

#### 4.7.2. Comparison with Other Representation Learning Methods

**Figure 8:**
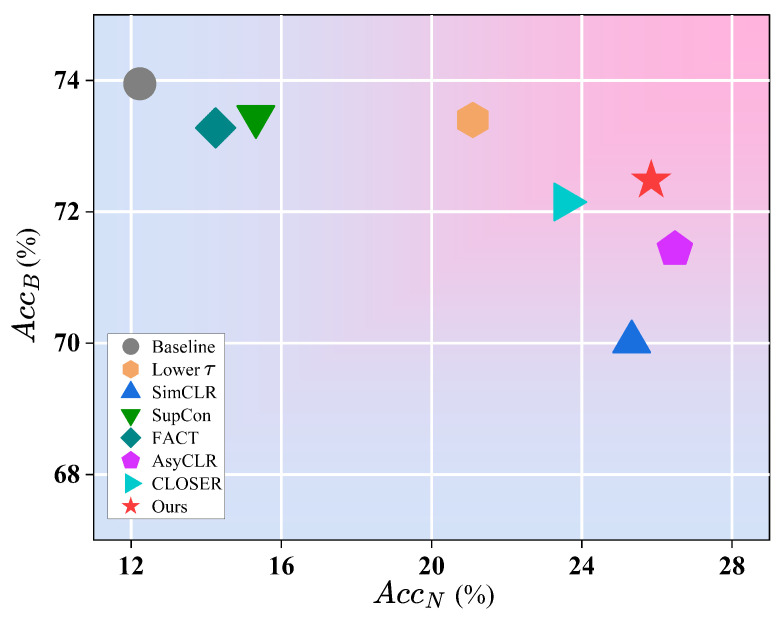
Performance comparison of different representation learning methods.

To further demonstrate the superiority of BR-FSCIL, we conduct a comprehensive comparison with other commonly used representation learning methods in FSCIL. These methods are all designed to learn powerful representations during the pre-training stage. Among them: Lower τ [29] employs a lower-temperature softmax cross-entropy loss for training. SimCLR [32] is a widely used self-supervised learning algorithm in FSCIL. SupCon [31] is a supervised contrastive learning algorithm. FACT [26] enhances the forward compatibility by generating virtual classes. CLOSER [16] compresses the feature space to obtain more balanced representations. AsyCLR [7] introduces predictive features to achieve asymmetric alignment of positive pairs, thereby mitigating excessive similarity within positive features. We set the training temperature τ to 1/16 for the baseline and to 1/32 for Lower τ. Previous studies have shown that the temperature parameter has a greater impact on representation transferability than the margin [16]. Therefore, the negative margin is not employed for comparison. Both SimCLR and SupCon losses are jointly trained with CE loss, omitting the non-linear projection heads. Figure 8 presents the classification accuracy of all compared methods in the final session on the mini-ImageNet. The vertical and horizontal axes represent the classification accuracy on novel and base classes, respectively. Thus, methods located closer to the top-right corner indicate better overall performance.

As shown in Figure 8, BR-FSCIL achieves the optimal balance between base-class and novel-class performance. In comparison, CE, SupCon, and FACT overemphasize base-class performance at the expense of model generalization. Lower τ improves the transferability of representations to some extent, yet still leaves considerable room for improvement, while SimCLR and AsyCLR significantly enhance model generalization to novel classes, they do so at the cost of base-class accuracy. Although CLOSER also demonstrates a relatively balanced performance, our method constructs a semantically richer feature space, thereby achieving superior performance on both base and novel classes. In summary, our method not only effectively enhances the transferability of representations but also maintains strong discriminability, resulting in the best results in FSCIL.

### 4.8. Visual Analysis

In this subsection, we present a visualization analysis of the proposed BR-FSCIL to intuitively demonstrate its effectiveness.

#### 4.8.1. GradCAM Visualization

**Figure 9:**
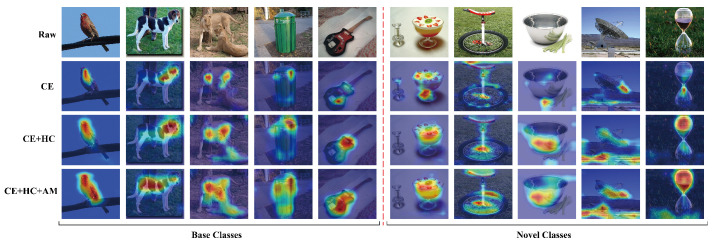
GradCAM visualization for base-class and novel-class exemplars.

To intuitively demonstrate the impact of each component on the learned representations, we perform a visual analysis using GradCAM [45] on five randomly selected base-class and five novel-class samples from mini-ImageNet. In the second row of Figure 9, when trained only with the CE loss, the baseline exhibits clear limitations in feature selection. The model’s attention is overly focused on local discriminative regions, while ignoring class-agnostic information that holds value for generalization. In contrast, after incorporating the HC loss from HierCon, the model’s attention expands significantly while still maintaining discriminative feature extraction for base classes. Furthermore, the model trained with the full loss (fourth row) demonstrates a stronger capability for global feature capture, as it consistently identifies transferable features across categories—such as biological posture, texture patterns, and other key visual cues. Notably, the baseline produces incomplete or incorrect feature localizations for novel classes, whereas BR-FSCIL achieves precise identification of their critical features. This comparison strongly confirms that our method enhances the model’s generalization and recognition robustness for novel objects.

#### 4.8.2. UMAP Visualization

**Figure 10:**
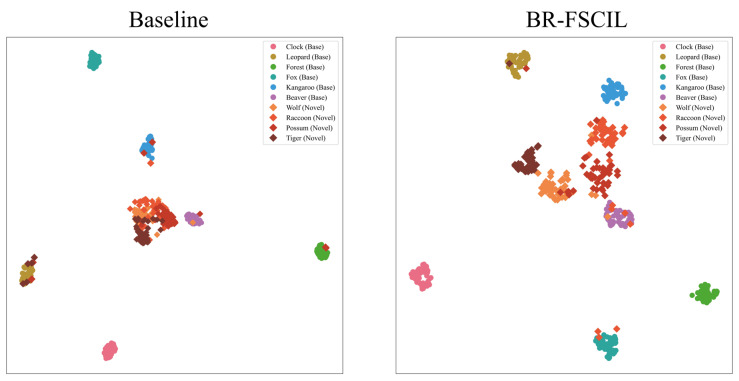
Visualization of the feature space using UMAP. Circles represent base classes, diamonds denote novel classes, and distinct colors indicate different subclasses.

We employ UMAP [46] to visualize the feature spaces of the baseline and our proposed BR-FSCIL. The experiment is conducted on the CIFAR100 dataset, using 50 samples from each of six randomly selected base classes and four novel classes for visualization. From Figure 10, although the baseline achieves satisfactory clustering of base-class samples by leveraging discriminative features, the excessive density of intra-class samples leads to class collapse. Consequently, the learned representations fail to effectively support clustering of novel-class samples, leading to significant overlap among features across different novel classes and ambiguous cluster boundaries. In contrast, the feature space learned by BR-FSCIL exhibits clear and compact cluster structures. Notably, our approach not only aggregates features within each base class but also mitigates excessive intra-class similarity, thereby enhancing the transferability of representations. This results in well-separated and distinct clusters for novel-class samples. Our feature space visualization strongly demonstrates the superiority of BR-FSCIL in balancing the discriminability and transferability of learned representations.

#### 4.8.3. Confusion Matrix Visualization

**Figure 11:**
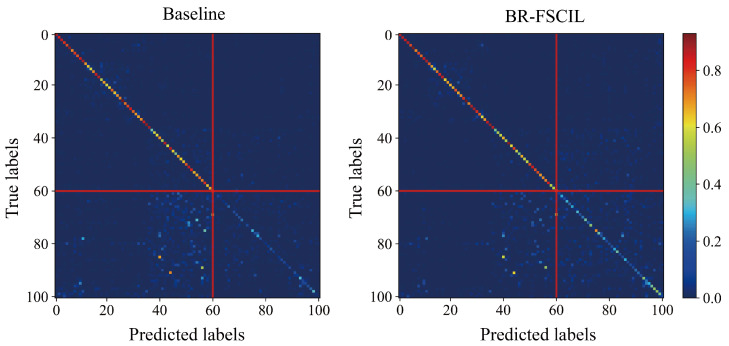
Confusion matrix visualization across all classes.

We also performed a visual analysis using confusion matrices. As depicted in Figure 11, both the baseline and BR-FSCIL exhibit bright base-class diagonals, indicating their strong performance on base classes. However, our BR-FSCIL demonstrates significantly higher brightness values along the diagonal corresponding to the novel classes. The results reveal that BR-FSCIL achieves notably superior performance in classifying novel classes while maintaining competitive base-class accuracy.

### 4.9. Computational Cost Analysis

To further demonstrate the practical value of our approach, we compare the computational overhead during training among three pre-training schemes on the mini-ImageNet dataset: the baseline, the baseline with SSCL, and our proposed BR-FSCIL. The evaluation metrics include GFLOPs per batch for forward propagation and the average training time per epoch. Following the experimental setup in Section 4.1.2, all methods use ResNet-18 as the backbone network with a batch size of 128. Since none of the methods introduce additional modules or employ sample replay, a single RTX 4090 GPU with 24 GB memory is sufficient for all experiments.

As shown in Table 9, the GFLOPs of the baseline with SSCL and BR-FSCIL are comparable, both approximately twice that of the baseline. This is because both SSCL and BR-FSCIL generate augmented views for each sample in the batch to facilitate contrastive learning, effectively doubling the number of processed samples per batch and leading to a significant increase in computational load. Additionally, contrastive learning introduces additional computations for measuring sample similarity. Nevertheless, by leveraging the GPU’s parallel computing capabilities, BR-FSCIL requires only about 50 s per epoch more than the baseline. It is worth noting that although the proposed loss function in BR-FSCIL introduces negligible extra floating-point operations compared to SSCL—as reflected in the nearly identical GFLOPs between the baseline with SSCL and BR-FSCIL—the additional loss computation still results in BR-FSCIL taking about 13 s longer per epoch than the baseline with SSCL. This overhead remains within an acceptable range. More importantly, once the BR-FSCIL model is pre-trained, no further fine-tuning is required in subsequent incremental sessions, nor are additional modules or memory for storing exemplars needed. Therefore, BR-FSCIL offers greater computational efficiency and practical advantages compared to other types of FSCIL methods.

## 5. Discussion

Our work focuses on addressing representation learning in FSCIL, aiming to learn representations that not only preserve the discriminability for known classes but also transfer effectively to unknown ones. During the incremental sessions, representations remain frozen to ensure stability. Although the representations are not updated with novel-class samples, plasticity is improved by enhancing transferability. Our method introduces no additional components to increase computational overhead. Instead, it optimizes representations during the pre-training phase to enhance model performance in incremental sessions, thereby providing a simple yet effective framework for FSCIL. However, although freezing representations during the incremental phase and using the NCM classifier with feature-average class prototypes mitigates catastrophic forgetting, the scarcity of novel-class samples leads to inaccurate prototypes, thereby limiting the model’s performance on novel classes. Consequently, optimizing classifier weights during the incremental learning stage will be a central focus of future research, which addresses the inaccuracy of feature-averaged class prototypes caused by extremely limited data to enhance the model’s adaptability.

## 6. Conclusions

In this paper, we observe that a unique challenge in FSCIL lies in achieving a balance between the discriminability and transferability of the learned representations. To address this issue, we propose BR-FSCIL. Specifically, we first introduce the Hierarchical Contrastive Learning algorithm, which captures hierarchical feature relationships and preserves discriminability during feature spread. Second, we design an Alignment Modulation loss to significantly improve transferability without compromising intra-class compactness. Our method effectively balances discriminability and transferability through joint optimization at both intra-class and inter-class levels, offering a simple yet effective representation learning framework designed for FSCIL. Extensive experiments on the mini-ImageNet, CIFAR100, and CUB200 datasets consistently demonstrate the effectiveness of our method in FSCIL scenarios, achieving final session accuracies of 53.83%, 53.04%, and 62.60%, respectively. Since our method maintains frozen representations during incremental sessions to prevent forgetting, a key direction for future research lies in optimizing classifier weights during the incremental learning phase, with the goal of further enhancing the model’s adaptability to novel concepts.

## Figures and Tables

**Table 1:** Summary of related works.

Fields	Type	Methods	Year	Key Features
Class-Incremental Learning	Regularization	EWC	2017	Proposes the adoption of the Fisher Information Matrix to safeguard critical parameters
		SOUL	2023	Reduces inter-class confusion within the Bi-GCN classifier
	Replay	iCaRL	2017	Adopts a class-prototype-based sample selection strategy to mitigate forgetting
		AAER	2022	Employs anchors as representative samples of prior classes
	Knowledge Distillation	LwF	2017	Employs a distillation loss to constrain shifts in the output space
		EEIL	2018	Proposes an end-to-end framework for incremental learning
	Fine-tuning	LRT	2024	Propose a novel fine-tuning-based paradigm to understand objects by joint visual clues and text depictions
Few-Shot Class-Incremental Learning	Fine-tuning	TOPIC	2020	Pioneers the FSCIL task and introduces Gas Neural Networks as its solution
	Pre-training	CEC	2021	Introduces a novel architecture that decouples the backbone from the classifier, employing graph attention for weight updates
		FACT	2022	Improve the model’s capability for forward compatibility
		SAVC	2023	Employs a more aggressive augmentation strategy in supervised contrastive learning
		CLOSER	2024	Emphasizes the significance of inter-class distance in the FSCIL scenario
		BFCP	2025	Further optimize the model’s forward compatibility in the FSCIL scenario
Representation Learning	Transferable Representation Learning	Lower Temperature	2021	Reducing the temperature parameter in the softmax cross-entropy loss to encourage feature sharing.
		Negative Margin	2022	Incorporating a negative margin into the softmax cross-entropy loss to promote feature sharing between classes
	Contrastive Learning	SimCLR	2020	Provides a simple yet effective Self-Supervised Contrastive Learning framework
		SupCon	2020	Incorporates label information into contrastive learning for the first time
		AsyCLR	2025	Introduces predictive features to implicitly align positive pairs

**Table 2:** Detailed statistics of three datasets.

	Mini-ImageNet	CIFAR100	CUB200
Total classes	100	100	200
Total samples	60,000	60,000	11,788
Base classes	60	60	100
Training samples per base class	500	500	30
Testing samples per base class	100	100	30
Novel classes	40	40	100
Incremental steps	8	8	10
Novel classes per step	5	5	10
Training samples per novel class	5	5	5

**Table 3:** Comparison on mini-ImageNet in the five-way five-shot FSCIL setting. The best results are shown in bold.

Method	Accuracy in Each Session (%)									Avg ↑	PD ↓
	**0**	**1**	**2**	**3**	**4**	**5**	**6**	**7**	**8**		
iCaRL (2017)	61.31	46.32	42.94	37.63	30.49	24.00	20.89	18.80	17.21	33.29	44.10
TOPIC (2020)	61.31	50.09	45.17	41.16	37.48	35.52	32.19	29.46	24.42	39.64	36.89
CEC (2021)	72.00	66.83	62.97	59.43	56.70	53.73	51.19	49.24	47.63	57.75	24.37
FACT (2022)	74.86	70.65	66.18	62.53	59.90	56.34	53.57	51.22	49.67	60.55	25.78
SAVC (2023)	**77.40**	**72.67**	68.41	64.37	61.10	57.71	54.79	52.61	50.90	62.22	26.50
SoftNet (2023)	76.63	70.13	65.92	62.52	59.49	56.56	53.71	51.72	50.48	60.79	26.15
UaD-CE (2023)	72.35	66.83	61.94	58.48	55.77	52.20	49.96	47.96	46.81	56.92	25.54
CLOSER (2024)	76.75	71.97	67.68	64.60	61.81	58.74	56.01	54.15	52.71	62.71	24.04
LIMIT (2024)	72.32	68.47	64.30	60.78	57.95	55.07	52.70	50.72	49.19	59.06	**23.13**
BFCP (2025)	77.15	71.54	66.93	63.65	60.58	57.81	55.47	53.41	51.48	62.00	25.67
BR-FSCIL (ours)	77.32	72.64	**68.77**	**65.71**	**62.65**	**59.86**	**56.93**	**55.05**	**53.83**	**63.64**	23.49

**Table 4:** Comparison on CIFAR100 in the five-way five-shot FSCIL setting. The best results are shown in bold.

Method	Accuracy in Each Session (%)									Avg ↑	PD ↓
	**0**	**1**	**2**	**3**	**4**	**5**	**6**	**7**	**8**		
iCaRL (2017)	64.10	53.28	41.69	34.13	27.93	25.06	20.41	15.48	13.73	32.87	50.37
TOPIC (2020)	64.10	55.88	47.07	45.16	40.11	36.38	33.96	31.55	29.37	42.62	34.73
CEC (2021)	73.07	68.88	65.26	61.19	58.09	55.57	53.22	51.34	49.14	59.53	23.93
FACT (2022)	75.93	72.26	67.75	63.80	61.45	58.23	56.09	53.97	51.82	62.37	24.11
SAVC (2023)	74.67	70.24	65.90	61.52	58.04	55.31	53.01	50.83	48.80	59.81	25.87
SoftNet (2023)	72.62	67.31	63.05	59.39	56.00	53.23	51.06	48.83	46.63	57.57	25.99
UaD-CE (2023)	75.55	71.78	65.47	62.83	55.56	55.08	50.11	46.35	40.46	58.13	35.09
CLOSER (2024)	74.50	70.69	67.40	63.51	60.87	57.98	56.09	54.15	51.85	61.89	22.65
LIMIT (2024)	73.81	72.09	67.87	63.89	60.70	57.77	55.67	53.52	51.23	61.84	**22.58**
BFCP (2025)	**76.43**	**72.49**	**68.63**	64.55	61.19	58.48	56.39	53.77	51.62	62.62	24.81
BR-FSCIL (ours)	76.18	72.35	68.42	**64.69**	**61.70**	**58.94**	**57.23**	**55.19**	**53.04**	**63.08**	23.14

**Table 5:** Comparison on CUB200 in the 10-way 5-shot FSCIL setting. The best results are shown in bold.

Method	Accuracy in Each Session (%)											Avg ↑	PD ↓
	**0**	**1**	**2**	**3**	**4**	**5**	**6**	**7**	**8**	**9**	**10**		
iCaRL (2017)	68.68	52.65	48.61	44.16	36.62	29.52	27.83	26.26	24.01	23.89	21.16	36.67	47.52
TOPIC (2020)	68.68	62.49	54.81	49.99	45.25	41.40	38.55	35.36	32.22	28.31	26.28	43.92	24.76
CEC (2021)	75.85	71.94	68.50	63.50	62.43	58.27	57.73	55.81	54.83	53.52	52.28	61.33	33.84
FACT (2022)	77.96	73.62	70.31	66.18	64.72	62.34	61.36	60.19	58.45	57.90	56.73	64.52	21.23
SAVC (2023)	79.99	76.62	73.94	69.96	68.20	65.34	64.17	62.57	60.83	60.39	59.63	67.42	20.36
SoftNet (2023)	78.07	74.58	71.37	67.54	65.37	62.60	61.07	59.37	57.53	57.21	56.75	64.68	21.32
CLOSER (2024)	**79.37**	**76.29**	**73.58**	**70.46**	**69.03**	66.89	66.16	64.74	62.95	62.97	62.13	**68.60**	17.08
LIMIT (2024)	75.90	73.23	70.84	66.13	65.56	62.15	61.74	59.83	58.41	57.89	56.94	64.42	18.96
BFCP (2025)	78.25	74.96	71.78	68.24	66.43	64.01	62.81	61.98	60.05	59.94	59.20	66.15	19.05
BR-FSCIL (ours)	78.74	75.80	73.11	70.23	68.98	**67.05**	**66.37**	**65.00**	**63.38**	**63.15**	**62.60**	68.58	**16.14**

**Table 6:** Ablation study on mini-ImageNet in the five-way five-shot FSCIL setting. The best results are shown in bold.

Components			Accuracy in Each Session (%)									Final Session		Avg ↑
**CE**	**HC**	**AM**	**0**	**1**	**2**	**3**	**4**	**5**	**6**	**7**	**8**	${\mathrm{Acc}}_{B}$	${\mathrm{Acc}}_{N}$	
✔			75.80	70.42	66.06	62.34	59.23	56.04	53.27	51.19	49.26	**73.95**	12.23	60.40
✔	✔		77.15	72.37	68.39	65.19	62.25	59.38	56.52	54.63	53.39	72.87	24.17	63.25
✔		✔	**78.06**	72.62	68.14	64.20	61.06	57.74	54.87	53.05	51.90	72.55	20.93	64.20
	✔		51.92	48.14	45.09	42.93	40.81	38.70	36.82	35.40	34.10	49.72	10.68	41.55
	✔	✔	19.38	17.72	16.64	16.59	15.43	14.71	14.01	13.45	12.98	15.15	09.72	15.66
✔	✔	✔	77.32	**72.64**	**68.77**	**65.71**	**62.65**	**59.86**	**56.93**	**55.05**	**53.83**	72.48	**25.85**	**63.64**

**Table 7:** Effect of the non-linear projection head on performance. The best results are shown in bold.

Loss	Final Session			Avg ↑
	Acc	${\mathrm{Acc}}_{B}$	${\mathrm{Acc}}_{N}$	
CE + HC w/o *g*	53.39	**72.87**	24.17	63.25
CE + HC w/*g*	52.08	71.23	23.37	61.19
CE + HC + AM w/o *g*	**53.83**	72.48	**25.85**	**63.64**
CE + HC+ AM w/*g*	51.41	70.42	22.90	60.76

**Table 8:** Comparison of different loss functions in key pattern alignment. The best results are shown in bold.

Loss	Final Session			Avg ↑
	Acc	${\mathrm{Acc}}_{B}$	${\mathrm{Acc}}_{N}$	
None	53.39	72.87	24.17	63.25
L1	53.48	72.25	25.33	63.50
Cosine Similarity	53.41	**73.08**	23.92	63.11
KL Divergence	53.46	72.72	24.57	63.36
Inter	53.55	71.70	**26.32**	62.82
AM (ours)	**53.83**	72.48	25.85	**63.64**

**Table 9:** Computational Cost Comparison.

Method	Backbone	GFLOPs	Training Time(s)
Baseline	ResNet-18	38.15	113
Baseline+SSCL	ResNet-18	76.34	150
BR-FSCIL	ResNet-18	76.34	163

## Data Availability

All datasets employed in this study are publicly available. These datasets can be accessed at mini-ImageNet: https://www.kaggle.com/datasets/zcyzhchyu/mini-imagenet/data (accessed on 16 September 2025), CIFAR100: https://www.kaggle.com/datasets/melikechan/cifar100/data (accessed on 16 September 2025) and CUB200: https://ieee-dataport.org/documents/caltech-ucsd-birds-200-2011#files (accessed on 14 September 2025).

## Abbreviations

The following abbreviations are used in this manuscript:

CIL	Class-Incremental Learning
FSCIL	Few-Shot Class-Incremental Learning
CE	Cross-Entropy
SSCL	Self-Supervised Contrastive Learning
SupCon	Supervised Contrastive Learning
HierCon	Hierarchical Contrastive Learning
AM	Alignment Modulation
